# Random and cyclical deletion of large DNA segments in the genome of *Pseudomonas putida*

**DOI:** 10.1111/j.1462-2920.2012.02730.x

**Published:** 2012-06

**Authors:** Audrey Leprince, Víctor de Lorenzo, Petra Völler, Mark W J van Passel, Vitor A P Martins dos Santos

**Affiliations:** 1 Systems and Synthetic Biology Group, Helmholtz-Centre for Infection Research38124 Braunschweig, Germany; 2 Laboratory of Systems and Synthetic Biology, Wageningen University6703HB Wageningen, the Netherlands; 3 Centro Nacional de Biotecnologia, Campus de Cantoblanco28049 Madrid, Spain

## Abstract

Cumulative site-directed mutagenesis is of limited suitability for the global analysis of the gene functions in the microbe's cellular network. In order to simplify and stabilize the genome of the soil bacterium *Pseudomonas putida*, we developed a recyclable three-step excision method based on the combination of customized mini-transposons and the FLP-*FRT* site-specific recombination system. To demonstrate the powerful potential of these tools, we first established insertion mutant libraries that allow users to study gene functions with respect either to phenotypic characteristics (single insertions) or to their involvement in predicted networks (double insertions). Based on these libraries, we generated as a proof-of-principle, single-deletion mutants lacking ∼ 4.1% of the genome (∼ 3.7% of the gene repertoire). A cyclical application of the method generated four double-deletion mutants of which a maximum of ∼ 7.4% of the chromosome (∼ 6.9% of the gene count) was excised. This procedure demonstrates a new strategy for rapid genome streamlining and gain of new insights into the molecular interactions and regulations.

## Introduction

The engineering of microbes at genome-scale holds the promise of both providing a thorough understanding of molecular interactions and quickly translating this knowledge into tailored applications of environmental, medical and industrial relevance (Warner *et al*., 2010; Woodruff and Gill, 2011). Extensive genetic modifications of microbes result in the alteration, insertion or elimination of, in most cases, one or a small number of genes at a time in a given strain (Wang *et al*., 2009). Although the pace at which this can be done is increasing rapidly, a fundamental hurdle for rational genome engineering remains the lack of knowledge of the microbial interactome.

Here we developed a multi-transposon mutagenesis method to rapidly eliminate large blocks of genes from a sizable microbial genome as a basis for genome streamlining. The tested bacterium, *Pseudomonas putida*, is a fast growing, ubiquitous and metabolically versatile soil microbe with an outstanding capacity to degrade a broad range of compounds, in particular aromatics, and of remarkable potential for biocatalysis (Jimenez *et al*., 2002; Dos Santos *et al*., 2004; Pieper *et al*., 2004; de Lorenzo, 2008). *Pseudomonas putida* KT2440 is a certified biosafety strain that was the first of the species to be sequenced (Nelson *et al*., 2002) and, by far, the most thoroughly and widely studied. It is an ideal host for heterologous gene expression (Gilbert *et al*., 2003; Dammeyer *et al*., 2011). The large gene pool and the entangled nature of the interactions, mostly unknown, between the various layers of regulation and metabolism, provide this bacterium with its remarkable resilience and versatility to thrive in harsh and different environments. Numerous data on the genetic, physiological and metabolic properties of the strain have been generated, including a genome-wide mutant library (Molina-Henares *et al*., 2009), transcriptomic, proteomic and metabolic datasets under a range of conditions (Kurbatov *et al*., 2006; Hervas *et al*., 2008), as well as experimentally validated genome-scale constraint-based models of metabolism and transport (Nogales *et al*., 2008; Puchalka *et al*., 2008). Global analyses of the genome, such as the G + C and the oligonucleotide contents, identified 105 genomic islands that may carry auxiliary functions (Weinel *et al*., 2002), as well as 184 proteins related to mobile elements, including among others 82 transposases, eight group II introns and three bacteriophages (Nelson *et al*., 2002; Dos Santos *et al*., 2004). However, despite the wealth of information and knowledge acquired thus far, about 30% of the genes encode unknown function and hypothetical proteins.Therefore, it is desirable to initiate the rational reprogramming of the organism, its tailoring for *à la carte* biocatalysis, and control and steering of the conceived functions, based on a genome whose products and interactions thereof are as simplified as possible to minimize the interferences with the metabolism and regulation of the cell. Therefore, we aimed to streamline the genome of *P. putida* by eliminating parts of the genetic machinery whose functions are dispensable under specific conditions.

**Fig. 1:**
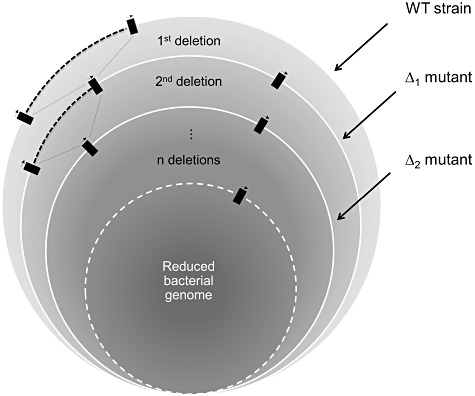
Cyclical random large-scale deletion method. The cyclical decrease in size of the white circles (not to scale) schematizes the reduction of the wild-type (WT) genome. Removal of random segments framed by recombinase recognition targets (black rectangles) successively generates the single-deletion (Δ_1_) and double-deletion (Δ_2_) mutants. Repetition of the method *n* times would produce a mutant reduced by *n* genomic fragments.

*Escherichia coli*, a workhorse of molecular biology, has been widely studied for targeted gene deletions, often based on the expression of site-specific recombinases (Yu *et al*., 2002; 2008; Goryshin *et al*., 2003). Also in *Pseudomonas* species, such as *P. aeruginosa* (Quénée *et al*., 2005) and *P. fluorescens* (Giddens *et al*., 2007), the commonly used Cre/*loxP* recombination system from phage P1 was applied. However, a homologous system, the FLP-*FRT* from *Saccharomyces cerevisiae*, was previously tested for the removal of previously inserted genetic elements in the strain KT2440 (De las Heras *et al*., 2008), and therefore chosen for the present work. In order to quickly remove a set of conditionally non-essential genes, we improved the deletion system. The method here described is a combinatorial method for streamlining *P. putida* based on the generation and use of customized mini-Tn*5* transposons combined with the FLP-*FRT* recombination system (Fig. 1) (Schweizer, 2003). The FLP recombinase is able to recognize two identical *FRT* sites (FLP Recognition Target) and, consequently, to excise the framed DNA fragment, when both sites are in the same orientation. Therefore, we employed the FLP as genetic scissors to delete various genomic fragments, of which the nature and size vary with the insertion and distance between both *FRT* sites. This method offers the possibility to gain unprecedented insights into the function of the genes and the metabolic pathways with the construction of insertion mutant libraries, as well as an improved way towards the categorization of conditionally essential and non-essential genes. As a proof-of-principle we generated a large library, from which we obtained four double-deletion mutants, deleting over two cycles up to 7.4% of a single genome.

## Results and discussion

### Mini-transposon libraries for *P. putida* strain TEC1

In order to randomly endow the strain with the genetic scissors, we customized two mini-transposons, mini-Tn*5 KpF* and mini-Tn*5 TF*, each carrying a single selectable marker [kanamycin resistance cassette (Km) and potassium tellurite degradation cassette (Tel) respectively] and one *FRT* site (Fig. S1). The sequence of each mini-transposon can be retrieved from GenBank under the following accession numbers: JQ406586 (mini-Tn*5 KpF*) and JQ406587 (mini-Tn*5 TF*). These mini-transposons are key elements for strain streamlining and are specifically engineered for an efficient isolation of the insertion mutants and a rapid generation of genomic deletions. The *pyrF* gene was used in mini-Tn*5 KpF* both as selectable and counter-selectable marker due to the lack of *pyrF* in TEC1 [uracil auxotroph and 5′-Fluoroorotic acid (FOA) resistant] (Galvao and de Lorenzo, 2005). Cloning of the 48 bp (base pairs) *FRT* sequence, into the mini-transposon derivatives, prepared the resulting insertion mutants for the recombination and deletion step.

**Fig. 2:**
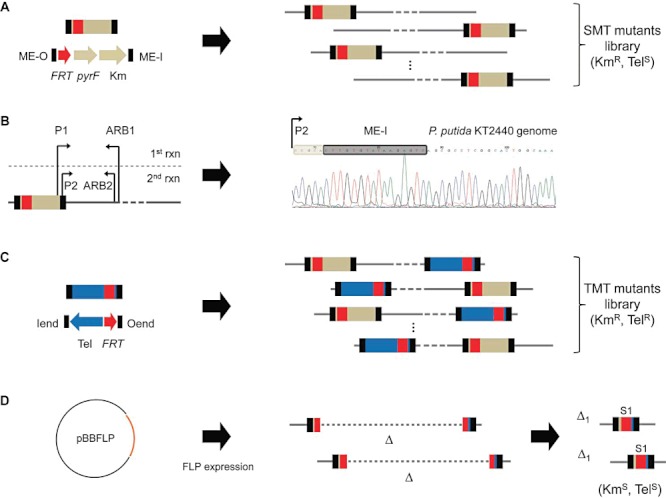
First random deletion in *P. putida*. A. Insertion of the mini-Tn*5 KpF* and generation of a library of Km^R^, Tel^S^ single mini-transposon (SMT) mutants. B. Mapping of the mini-Tn*5* by AP-PCR for each SMT mutant, following two rounds (rxn) of amplification and using two pairs of primer (P1/ARB1 and P2/ARB2). Sequencing of the PCR products, using P2. The results were blasted against the *P. putida* KT2440 genome. C. Insertion of the mini-Tn*5 TF* into selected SMT candidates and generation of a library of Km^R^, Tel^R^ two mini-transposon (TMT) mutants. Mapping of mini-Tn*5*s by applying AP-PCR as in B. D. Insertion of pBBFLP vector and expression of the FLP recombinase in the selected TMT candidates. Selection of the Km^S^, Tel^S^ single-deletion (Δ_1_) mutant.

After transposition of mini-Tn*5 KpF* to TEC1, 61 single mini-transposon (SMT) mutants (93% of the tested colonies) were isolated on minimal medium (Fig. 2A). Mapping the mini-Tn*5 KpF* for each mutant by arbitrarily primed-polymerase chain reaction (AP-PCR) revealed a different location in ∼ 90% of the cases (Figs 2B and S2A). Fifty-one genes were disrupted, covering 15 of the 20 cellular role categories found in TEC1, and three insertions occurred in distinct intergenic regions (data not shown). We next chose randomly nine SMT mutants (Table 1) for insertion of mini-Tn*5 TF* and generation of a two mini-transposon (TMT) mutant library. We selected 573 Km^R^, Tel^R^, FOA^S^ and Piperacillin (Pip)^S^ TMT mutants, corresponding to 93.5% of the tested colonies (Fig. 2C) and submitted half of this library to AP-PCR to determine the location of each pair of mini-Tn*5* derivatives. In 90% of the cases, a different gene or intergenic region was disrupted by a mini-Tn*5 TF*, which represented 255 independent hits spread over the chromosome (Fig. S2B).

**Table 1:** Description of the disrupted genes in the selected SMT and further TMT mutants

Mutant ID	Gene	Function of the disrupted gene
SMT		
1	PP_5025 (mdoH)	Glucosyltransferase MdoH
2	PP_1695	Integral membrane sensor hybrid histidine kinase
3	PP_3490	Cupredoxin domain protein
5	PP_1490	Methyltransferase, CheR-like
6	PP_1869	Extradiol dioxygenase
7	PP_3534	LysR family transcriptional regulator
8	PP_4828 (cobH)	Precorrin-8X methylmutase
9	PP_0077 (betC)	Choline sulfatase
10	PP_0072 (qor-1)	Quinone oxidoreductase
TMT		
21 (9)	PP_0168 (lapA)	Large adhesion, surface associated
69 (10)	PP_0179	RND efflux transporter
91 (3)	PP_3529	Ssud-like monooxygenase
113 (9)	PP_0971	Conserved hypothetical protein
253 (3)	PP_4011 (icd)	Isocitrate dehydrogenase, NADP-dependent
407 (7)	PP_3733	ABC transporter lipoprotein, putative
475 (10)	IR_5275–5276	–

IR, intergenic region.

The mini-transposon insertions in the SMT and TMT mutants highlighted the non-essentiality of the genes that were disrupted individually or in combinations, when grown in minimal medium supplemented with citrate and uracil. We also compared this list of genes with the list of persistent genes established in *P. putida* F1 (A. Danchin, pers. comm.), highly similar to strain KT2440. A persistent gene was described as being present in a majority of organisms. The persistence does not necessarily imply the conditional essentiality of a gene while a non-essential gene does not automatically induce its non-persistence (Fang *et al*., 2005; Danchin, 2009). In our study, we found six genes considered as persistent which were hit by a mini-Tn*5* derivative: PP_0245, encoding a S1 RNA-binding domain-containing protein; PP_0483, encoding an excinuclease ABC subunit A; PP_0806, encoding a seed colonization adhesion protein LapF; PP_1896, encoding an ABC transporter; PP_4111, encoding an elongation factor G; and PP_4185, encoding a succinyl-CoA synthetase subunit alpha. These six genes illustrate properly the case of a persistent gene coding for non-essential function for the survival of the cell under experimental conditions.

### First round of deletions

The double-insertion mutants library representatively provide TEC1 with a range of targets for the FLP recombinase. As a proof-of-principle we pre-selected, *in silico*, potential candidates for deletion. We first screened the TMT mutants to choose the ones in which both *FRT* sites were inserted in the same orientation (Schweizer, 2003). Additionally, we considered the relative order of both mini-Tn*5* derivatives within the chromosome leading to the simultaneous loss of the resistance cassettes and the framed genomic fragment. The corresponding single-deletion mutants (Δ_1_) would retrieve the wild-type phenotype and would further be used for the next round of deletion. Following this approach, we determined 63 mapped TMT candidates (24.8%) leading to the expected Km^S^, Tel^S^, FOA^R^ phenotype. We analysed them in greater details and selected the candidates that would generate a broad-ranged size of deletion [i.e. between 41 and ∼ 1000 kb (kilobase pairs)]. We kept apart the candidates in which the origin of replication as well as predicted conditional essential genes might be lost after deletion in order to maintain a certain fitness of the cells (Nogales *et al*., 2008; Puchalka *et al*., 2008; Molina-Henares *et al*., 2010).

In total, seven TMT mutants were selected, issued from four independent SMT mutants (Table 1), for the conjugative introduction and expression of the recombinase (Fig. 2D). After selection on sucrose Lysogeny-broth (LB) medium, two independent Km^S^, Tel^S^, FOA^R^ and tetracycline (Tc)^R^ single-deletion mutants (29% of the tested TMT mutants) were generated: *P. putida* TEC1 91-Δ_1_ and 407-Δ_1_, issued from 91-TMT and 407-TMT respectively. The size of the lost fragments corresponded to 0.67% (41.5 kb) and 4.1% (253.9 kb) of the chromosome length respectively, which we predicted *in silico* based on the position of the mini-Tn*5* derivatives in the TMT mutants and we validated by PCR experiments with the Δ_1_ mutants. The full list of deleted genes is provided in Tables S1 and S2. Sequencing the scar (S1) confirmed the rearrangement between both *FRT* sites as well as the absence of mutations. Its sequence revealed segments from the mini-Tn*5* derivatives and the recombined *FRT* site, and was flanked by distant genomic DNA sequences (Figs 2D and S3). It was interesting to notice the proximity between the excisions in the two independent single-deletion mutants. Only four genes exist between the end of the deletion in 91-Δ_1_ (PP_3529) and the beginning of the one in 407-Δ_1_ (PP_3534). We did not find any persistent gene predicted *in silico* to be transcribed in these areas. We further streaked the mutant strains on M9 plates supplemented with uracil and citrate in order to assess the conditional essentiality of the group of deleted genes. TEC1 407-Δ_1_ grew in both LB and M9 media, supplemented with citrate and uracil. Mutant 91-Δ_1_, however, was able to grow only in LB medium. Comparison with the list of genes predicted essential for growth in M9 medium, with glucose as carbon source, revealed the potential responsibility of the branched-chain amino acid (BCAA) aminotransferase (IlvE), encoded by PP_3511 (*ilvE*) (Nogales *et al*., 2008). Supplementation of the minimal medium with the three BCAA (valine, leucine and isoleucine) was enough to restore growth of the mutant. Complementation of the 91-Δ_1_ mutant with the *ilvE* gene and assessment of its growth profile (data not shown) finally confirmed the essential role of the aminotransferase for growth in minimal medium, also when supplemented with citrate as carbon source.

### Second and successive genomic deletions

**Fig. 3:**
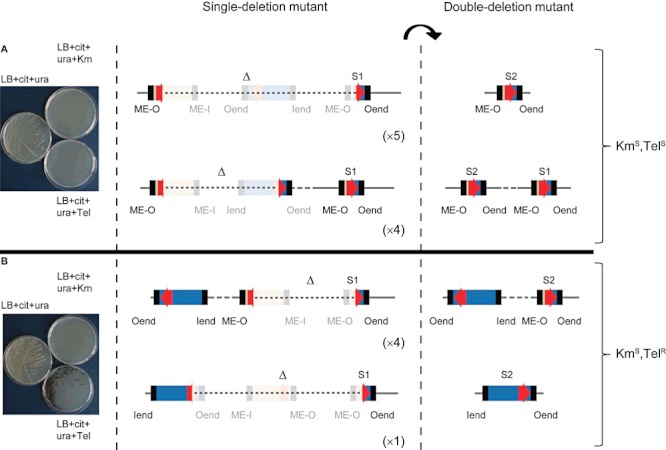
Possible configurations for the scar S2. A. Left: observed phenotype for 407.1-Δ_2_ double-insertion mutant. Possible orientation (red arrow) and position of the newly inserted mini-Tn*5*s and the scar S1 in the Km^S^, Tel^S^ single-deletion mutant. Two cases only are reported to symbolize the nine possible different situations, depending on the orientation and position of the passive *FRT* site. Right: visualization of the resulting scar S2 in the double-deletion mutant, either as a single scar or in the presence of S1 (both identical in sequence). B. Left: observed phenotype for 407.3-Δ_2_ mutant. Possible orientation and position of the newly inserted mini-Tn*5*s and S1 in the Km^S^, Tel^R^ single-deletion mutant (five different cases in total). Right: visualization of the resulting S2 in the double-deletion mutant, either in presence of mini-Tn*5 TF* or as a single scar. *FRT* sites (red arrow), Km resistance cassette (beige), Tel degradation cassette (blue), ends of mini-Tn*5*s (black), respectively, Oend/Iend and ME-O/ME-I.

As a result of the first genomic excision, S1 retains a single *FRT* site. Considering that a minimum of two sites is necessary for deleting a genomic fragment, we inserted both mini-Tn*5*s into 91-Δ_1_ and 407-Δ_1_ mutants, multiplying the targets for recombination (Fig. 1). Mutants Δ_1_ SMT and further Δ_1_ TMT were pooled after each triparental mating, omitting the intermediary mapping of the mini-transposons, and were directly used for the insertion and expression of the recombinase, therefore increasing the amount of candidates for a second deletion. The recombinase carrying vector was finally transferred to a pool of Δ_1_ TMT mutants featured with three integrated *FRT* sites. After the recombination and deletion step, putative double-deletion mutants (91-Δ_2_ and 407-Δ_2_) were tested for resistance to Tel, Km, Pip and FOA on LB plates. We found 26% of the putative 91-Δ_2_ mutants to be Pip^S^ and Km^S^ but Tel^R^. For putative 407-Δ_2_ mutants we found 62% that were Pip^S^ and Km^S^, an equal share was either Tel^R^ or Tel^S^. The loss of at least one resistance feature implied a genomic rearrangement leading to a second deletion in both 91-Δ_2_ and 407-Δ_2_. We isolated therefore colonies with either phenotype. We drew *in silico* an overview of the possible genomic rearrangements and subsequent scars present in the Δ_1_ TMT and resulting Δ_2_ mutants (Fig. 3). Considering the phenotypes observed (Km^S^ and Tel^R^ or Km^S^ and Tel^S^) and the orientation and position taken by the mini-Tn*5 TF* and *KpF*, we distinguished two configurations for the new scar (S2) in terms of sequence in the Δ_2_ mutants and four possible scenarios.

### Double-deletion Δ_2_ mutants

**Fig. 4:**
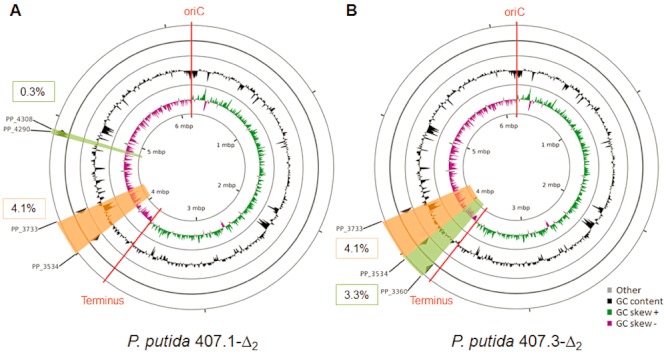
Genomic deletions in the double-deletion mutants of *P. putida*. The oranges boxes represent the 4.1% loss in the genome of the 407-Δ_1_ single-deletion mutant. The green boxes highlight the different second genomic deletions in the 407.1-Δ_2_ (A), corresponding to distant loss of 0.3% and 407.3-Δ_2_ (B) mutants, corresponding to an adjacent loss of 3.3% of the genome size. The origin (oriC) and terminus of replication are indicated in red. The maps were generated using the CGView genome viewer (Grant and Stothard, 2008).

The potential remaining presence of S1 in the Δ_2_ mutants was verified by PCR. We selected five (Km^S^, Tel^R^) 91-Δ_2_, five (Km^S^, Tel^R^) 407-Δ_2_ and five (Km^S^, Tel^S^) 407-Δ_2_ mutants to test this approach. Experiments with the latest indicated the remaining presence of the *FRT* site from S1 and, therefore, the recombination between the newly inserted mini-transposons (Fig. 3A, presence of S1 and S2 in the chromosome). The rearrangement between the *FRT* sites was verified by genome *de novo* sequencing (BaseClear, Leiden, the Netherlands) that revealed the lack of 19 additional consecutive genes for mutant 407.1-Δ_2_ (Table S3), located more than 600 kb downstream of the first deletion (Fig. 4A). Combined, this corresponded to a reduction of 4.4% of the genome size (219 lost genes). In the case of (Km^S^, Tel^R^) 91-Δ_2_ and 407-Δ_2_ mutants, *FRT* sites present in S1 and in mini-Tn*5 KpF* seemed to have recombined and hence deleted a fragment adjacent to the first deletion (Figs 3B and 4B). By direct genome sequencing (BaseClear), we found that the second deletion in the chromosome of 407.3-Δ_2_ (Km^S^, Tel^R^) excised 174 additional genes (Table S4), extending the whole deletion to 7.4% of the chromosome, which is a technical advance in rapid large-scale deletions. In total, the mutant lacked 372 consecutive protein-coding ORFs (6.9% of the total gene count) including among others 117 hypothetical protein coding genes and seven transposase genes (∼ 8% of the set), covering four of the eight paralogous families present in *P. putida*. The deletion of the latest may contribute to genetically stabilize the strain, as it was shown in the past with the generation of mutants from *E. coli* in which the insertion sequences were deleted increasing the genetic stability (Pósfai *et al*., 2006). The two Δ_2_ mutants generated in the present study revealed the diversity in the size range of mutation combinations that one can obtain by applying this procedure. We were able to expand the size of the first large deletion from 407-Δ_1_ to obtain 407.3-Δ_2_ mutant but also to create a second deletion much smaller at a different site in the chromosome of 407.1-Δ_2_ mutant. Both second deletions were located in areas in which persistent genes were predicted to be absent and only four genes were predicted as essential when grown in M9 medium (Nogales *et al*., 2008) supplemented with glucose as carbon source. Time-dependent cell density was measured for the single- and double-deletion mutants in LB medium; all showed a similar or better growth than the wild-type, with final cell densities up to 1.4 times higher than TEC1. Double-deletion mutants obtained from 91-Δ_1_ mutants were deprived of *ilvE* gene and were not able to grow in minimal medium without the supplement of BCAA. This predicted essential gene was the only one verified in the case of growth with citrate. The three other predicted essential genes for growth in glucose within these areas (PP_3363, PP_3633 and PP_3721) did not seem to play any essential role in the growth with citrate when simultaneously deleted (PP_3633 and PP_3721) in 407-Δ_1_ and (PP_3363, PP_3633 and PP_3721) in 407.3-Δ_2_. Observing the preference of the strain to delete these parts of the genome, it is interesting to correlate the possible random deletion with certain favourite genomic islands.

Finally, full genome sequencing of both Δ_2_ mutants gathered precious information for future analyses; however, it would be possible to further enhance the detection of the second deletion by applying appropriate modifications to the mini-Tn*5* transposons.

### Conclusions

The work herein described reports the first random large-scale deletion in a *Pseudomonas* species, and one for which no prior knowledge of potential dispensable genes is required, and a new step in genome streamlining. This method differs substantially from previous genome reduction approaches in *E. coli* (Pósfai *et al*., 2006; Yu *et al*., 2008) and *P. putida* (Martinez-Garcia and de Lorenzo, 2011), due to our rapid generation of insertion libraries from which numerous genomic regions can be deleted over successive rounds of reduction. The possible multiplexing and automation of part of the streamlining process could greatly accelerate and facilitate the workflow.

The repetition of the procedure on the double-deletion mutants as well as the establishment of further multiple-deletion (Δ_x_) SMT mutants could allow further increases in the genome reduction beyond the 7.4% already obtained, leading towards determining a functional essential core of the genome under the specified conditions. Through detailed ‘omics’, genetic and biochemical analyses, this would give invaluable insights into the metabolic and regulatory pathways of this fascinating bacterium, it would increase our knowledge about gene essentiality and, more importantly, of the interactions among genes, which cannot be predicted on the basis of annotated functions.

Once adapted, validated and applied to other relevant bacteria, this method could open new avenues for the streamlining of the genomes of Gram-negative bacteria, due to the use of the broad host range vectors pBAM1 and pBBFLP. This could provide precious tools to unravel metabolic and regulatory networks, the function and nature of the interactions of the genes and to, subsequently engineer genomes for enhanced *à la carte* biocatalysis in industrial contexts and in the bioremediation process of contaminated sites.

## Experimental procedures

### Bacterial strains and media

*Escherichia coli* and *P. putida* strains were grown at 37°C and 30°C respectively, in LB medium supplied with the following antibiotics and mineral compound when described: chloramphenicol (Cm) 15 µg ml^−1^, gentamicin (Gm) 30 µg ml^−1^, kanamycin (Km) 50 µg ml^−1^, piperacillin (Pip) 40 µg ml^−1^, potassium tellurite (Tel) 40 µg ml^−1^ and tetracycline (Tc) 15 µg ml^−1^. 5-Fluoroorotic acid (FOA, Zymo Research) was added to the media at 350 µg ml^−1^. For the counter-selection of *E. coli* strains, nalidixic acid (8 µg ml^−1^) was added to LB. Additionally, *P. putida* TEC1 was grown in M9 minimal medium supplemented with citrate (2 g l^−1^) as the carbon source and uracil (20 µg ml^−1^) (Galvao and de Lorenzo, 2005). We carried out the restriction and ligation (T4 DNA Ligase) procedures following the provider's instructions (New England Biolabs, Fermentas and Roche). For chemical transformations we transferred ∼ 400 ng of plasmids to 100 µl of *E. coli* competent strains following the standard method (O'Toole *et al*., 1999). We isolated and purified genomic DNA of the mutant strains using the Wizard Genomic DNA Purification Kit (Promega). Primers for the different PCR and AP-PCR experiments were synthesized by Eurofins MWG Operon and Eurogentec. We purified all PCR products with the QIAquick PCR Purification Kit (QIAGEN).

### Construction of mini-Tn*5* derivatives

We generated mini-Tn*5 KpF* and mini-Tn*5 TF* based on pBAM1 (Martinez-Garcia *et al*., 2011) and pJMT6 (Sanchez-Romero *et al*., 1998) respectively (Leprince *et al*., 2012). Briefly, the *pyrF* operon was amplified from the genomic DNA of *P. putida* KT2440 and flanked with BamHI and HindIII sites using the *pyrF*1F and *pyrF*2R primers. The fragment was further cloned into BamHI/HindIII sites of patt*FRT* vector (De las Heras *et al*., 2008) downstream of the *FRT* site. We then amplified the whole fragment *FRT*::*pyrF* with the primers Fp 1F and Fp 2R which added NotI restriction site at both ends and inserted in pBAM1 at the corresponding sites. In order to construct mini-Tn*5 TF*, we amplified the *FRT* fragment with the primers F 10F and F 11R and cloned it into the pGEM®-T Easy vector (Promega, Madison). We further extracted the *FRT* fragment by restriction with NotI and cloned it into pJMT6. The primer sequences can be found in Table S5. The generated delivery vectors were named pBAM1-*KpF* (mini-Tn*5 KpF*) and pJMT6-*TF* (mini-Tn*5 TF*).

### Chromosomal insertion of mini-Tn*5 KpF* and mini-Tn*5 TF*

We first transferred both suicide mini-Tn*5* derivative carrying plasmids to competent *E. coli* strain CC118λ*pir* by chemical transformation. We inserted then pBAM *KpF* into *P. putida* TEC1 cells by triparental mating (De Lorenzo and Timmis, 1994). Exconjugants were selected on M9 medium supplemented with citrate and Km. In order to confirm the loss of the delivery plasmids and therefore, to avoid new transposition events; we confirmed the true transposition for each colony by verifying their sensitivity to Pip and FOA. We used a pool of nine SMT mutants as acceptor strains for the insertion of mini-Tn*5 TF* by triparental mating using *E. coli* CC118λ*pir* pJMT6 *TF* as donor. We selected exconjugants on M9 medium supplemented with citrate, Km and Tel and verified the true transposition events by screening for a Pip^S^ phenotype.

### Presence and uniqueness of the mini-Tn*5* insertion

We first verified the uniqueness of the mini-Tn*5* insertion by Southern blot analyses. We digested 4 µg of genomic DNA with EcoRV and separated the restriction mixture on a 0.8% (wt/vol) agarose gel. We further transferred the embedded fragmented gDNA overnight to a positively charged Nylon Membrane (Hyperbond N+, Amersham). In the mean time we amplified by PCR the kanamycin and tellurite probes from the Km resistance gene (mini-Tn*5 KpF*) and from the Tel cassette (*telA* gene from mini-Tn*5 TF*) respectively, with the following sets of primers: Km-pBAM-F/Km-pBAM-R and Tel-F/Tel-R. The primers sequences are found in Table S6. The probes were labelled, following the provider's instructions (Amersham) and mixed with the buffer containing the membrane for overnight hybridization at 42°C. After washing the membrane and applying the detection reagents, homologous sequences inside the restricted fragments were revealed by chemiluminescence.

### Identification of the chromosomal mini-Tn*5* insertions

We then mapped the position of the inserted mini-Tn*5*s by AP-PCR using the genomic DNA of the SMT and TMT mutants (Fig. 2B). The detailed method is described elsewhere (Leprince *et al*., 2012). Briefly, two pairs of primers were used in two separated rounds of amplification with different sequences depending on the targeted mini-Tn*5* transposons (Table S7). We applied some modifications to the method. The P2 primer used for analysing mini-Tn*5 TF* was replaced by F 10F. The volume reactions corresponded to half of the described volumes and the annealing step for the 30 cycles was 50°C for 30 s, for the first round, and 56°C for 30 s, for the second round. Purified PCR products were sent for sequencing (Eurofins MWG Operon) with the external primer P2. The results were analysed with the Sequence Scanner v1.0 software (Applied Biosystems) and compared with the genome of *P. putida* KT2440 using the blastn program (NCBI).

### Deletion of the first genomic fragments

We transferred the pBBFLP vector via triparental mating to each TMT candidate. The exconjugants were selected on M9 medium supplemented with cit and Tc. Inoculation of the Tc^R^ exconjugants in LB supplemented with cit, ura and Tc, allowed expression of the FLP and consequent recombination between the two *FRT* sites present in the genome. Further selection on 5% (wt/vol) sucrose medium allowed the elimination of pBBFLP from the cells after recombination. Successful deletion events were determined by streaking up to several hundred colonies onto basic citrate and uracil media and Km, Tel or FOA supplemented media. PCR experiments were carried out with the Tel^S^, Km^S^ and FOA^R^ colonies. Knowing the original position of the two mini-transposons for each TMT candidate we designed specific primers to amplify a 400–800 bp product in each successful corresponding Δ_1_ mutant with Primer3-web (v. 0.4.0) interface of the Primer3 software (Rozen and Skaletsky, 2000). The different PCR products were sequenced at Eurofins MWG Operon with both primers. The sequencing results were blasted against the genome of *P. putida* KT2440 (Fig. S3).

### Second random genomic deletion

The mini-Tn*5 TF* was inserted by conjugation in the genome of the Δ_1_ mutants and the exconjugants were selected on LB plates containing citrate, uracil, Tel and Na. Putative Δ_1_ SMT mutants were directly resuspended from the plates with LB containing citrate and Tel and used as acceptor strain for the insertion of mini-Tn*5 KpF*. We selected the putative Δ_1_ TMT mutants on LB agar supplemented with citrate, Tel, Km and Na. We resuspended directly the exconjugants with the corresponding LB broth. After overnight incubation, the culture was involved as acceptor strain in the conjugation with *E. coli* HB101 and *E. coli* CC118λ*pir* pBBFLP. We allowed the FLP carrying plasmid to replicate and express the recombinase overnight on medium containing citrate, uracil, Na and Tc. We further gathered colonies together and inoculated them in LB supplemented with uracil, citrate, Na and Tc. After a short incubation the culture was spread on 5% sucrose medium. An average of 2–3 days at 30°C was necessary to isolate colonies which were tested on LB supplemented with citrate and uracil and containing either Km, Tel, FOA or Pip. Verification of the deleted fragments were carried either with genome *de novo* or direct genome sequencing experiments. In any case bacterial material (single colonies on LB plates) was sent for genomic DNA isolation (BaseClear, Leiden, the Netherlands). Genome *de novo* sequencing was further processed by the company as a paired-end sequencing on an Illumina platform over 50 runs delivering reads of 75 bp. Sequencing data were analysed with the Tablet graphical viewer (Milne *et al*., 2010). Direct sequencing was carried out using the primer PP_3733 rv (5′-AGGGCCAGGCTTCGTACTAT), designed from the genomic DNA fragment located downstream of the scar S1 and orientated towards the Oend of S1. Sequencing results were blasted against the genome of *P. putida* KT2440.

## Supporting information

**Fig. S1.** Distribution of the custom mini-Tn*5* positions in the insertion mutants.

A. Map of the mini-Tn*5 KpF* carrying pBAM1-*KpF* vector. The mini-Tn*5* is framed by the ends of the mini-transposon (ME-I and ME-O), represented by the gray boxes.

B. Map of the mini-Tn*5 TF* carrying pJMT6-*TF* vector. The mini-Tn*5* is framed by the ends of the mini-transposon (I and O ends), represented by the gray boxes. The *FRT* sites are represented by the short red arrow. The pink arrows outside the map indicate the binding site of the corresponding primers, and their orientation.

**Fig. S2.** Distribution of the custom mini-Tn*5* positions in the insertion mutants.

A. Map of the mini-Tn*5 KpF* insertions in the gathered single mini-transposon (SMT) mutants and overview of their distribution. The different hits are combined on the same graphical map for the convenience of the representation. Selection of nine SMT mutants for the insertion of the mini-Tn*5 TF* transposon (Table 1).

B. Map of the mini-Tn*5 TF* insertions in the analysed two mini-transposon (TMT) mutants and overview of their distribution over the chromosome. GC content (black circle), fluctuation of the GC skew: positive (green) and negative (Lila) values.

**Fig. S3.** Verification of the deletion event. Amplification of the scar S1 (underlined in green) left after the single deletions and sequencing of the PCR products are presented here.

A. The scar, composed of the end sites (gray) and internal fragments (orange) of the mini-Tn*5* transposons and one *FRT* sequence (red), was formed in 91-Δ_1_ mutant between the disrupted PP_3490 and PP_3529 genes.

B. The same scar was formed in 407-Δ_1_ mutant between the disrupted PP_3534 and PP_3733 genes. The sequencing confirmed the absence of mutation in the *FRT* fragment, after alignment with the two mini-Tn*5* transposons in A and B. The red rectangle represents the recombination point between both *FRT* sites present in the genome of TMT mutant before deletion.

**Table S1.** Deleted genes in 91-Δ_1_ mutant.

**Table S2.** Deleted genes in 407-Δ_1_ mutant.

**Table S3.** Second deletion in 407.1-Δ_2_ mutant.

**Table S4.** Second deletion in 407.3-Δ_2_ mutant.

**Table S5.** Primers for the customized mini-transposons.

**Table S6.** Primers for Southern blot experiments.

**Table S7.** Primers for AP-PCR experiments.
