# Supplementary material for: Random and cyclical deletion of large DNA segments in the genome of Pseudomonas putida
Source: Environ Microbiol. 2012 Jun;14(6):1444–53. doi: 10.1111/j.1462-2920.2012.02730.x (PMC3429869; doi:10.1111/j.1462-2920.2012.02730.x)
Supplement: Supplementary file 1 [file emi0014-1444-SD1.doc]

Supplementary Information for:

# Random and cyclical deletion of large DNA segments in the genome of *Pseudomonas putida*

### Audrey Leprince^1,3^, Víctor de Lorenzo^2^, Petra Völler^1^, Mark WJ van Passel^3^, and Vitor AP Martins dos Santos^1,3^


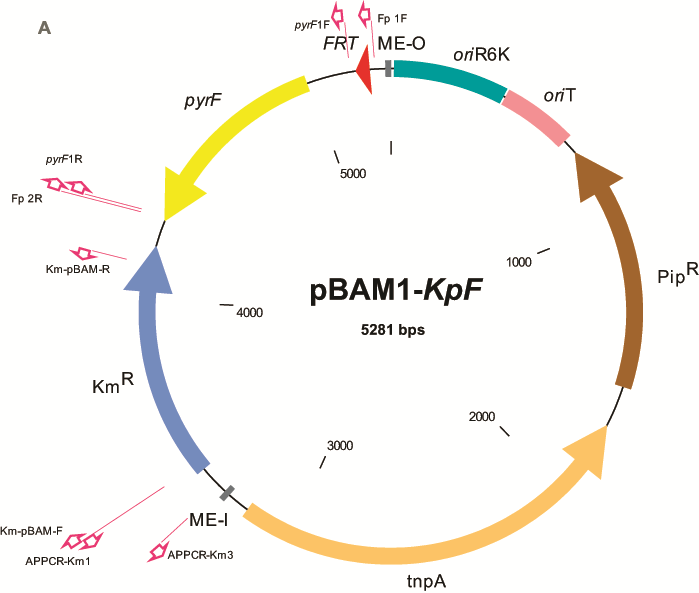


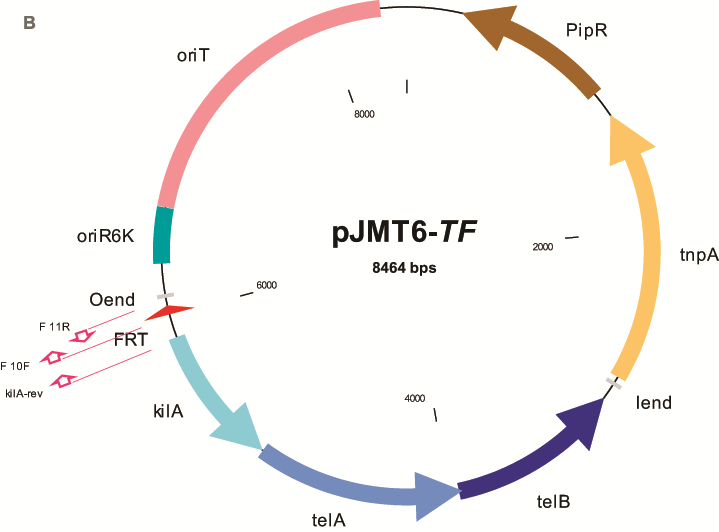


**Supplementary Figure S1 - Distribution of the custom mini-Tn*5* positions in the insertion mutants**

(**A**) Map of the mini-Tn*5 KpF* carrying pBAM1-*KpF* vector. The mini-Tn*5* is framed by the ends of the mini-transposon (ME-I and ME-O), represented by the gray boxes. (B) Map of the mini-Tn*5 TF* carrying pJMT6-*TF* vector. The mini-Tn*5* is framed by the ends of the mini-transposon (I and O ends), represented by the gray boxes. The *FRT* sites are represented by the short red arrow. The pink arrows outside the map indicate the binding site of the corresponding primers, and their orientation.


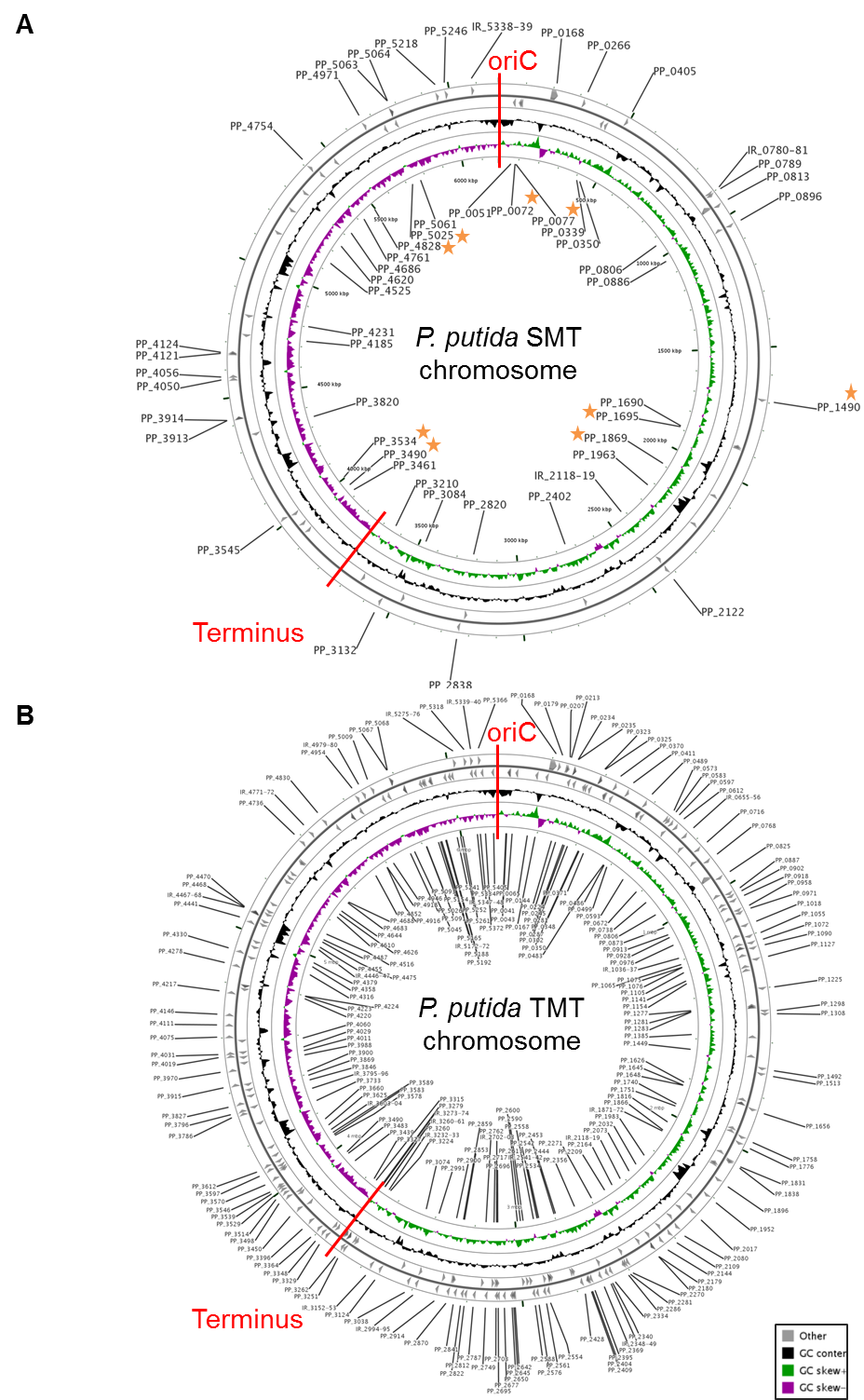


**Supplementary Figure S2 - Distribution of the custom mini-Tn*5* positions in the insertion mutants**

(**A**) Map of the mini-Tn*5 KpF* insertions in the gathered single mini-transposon (SMT) mutants and overview of their distribution. The different hits are combined on the same graphical map for the convenience of the representation. Selection of nine SMT mutants for the insertion of the mini-Tn*5 TF* transposon (Table 1). (**B**) Map of the mini-Tn*5 TF* insertions in the analyzed two mini-transposon (TMT) mutants and overview of their distribution over the chromosome. GC content (black circle), fluctuation of the GC skew: positive (green) and negative (Lila) values.


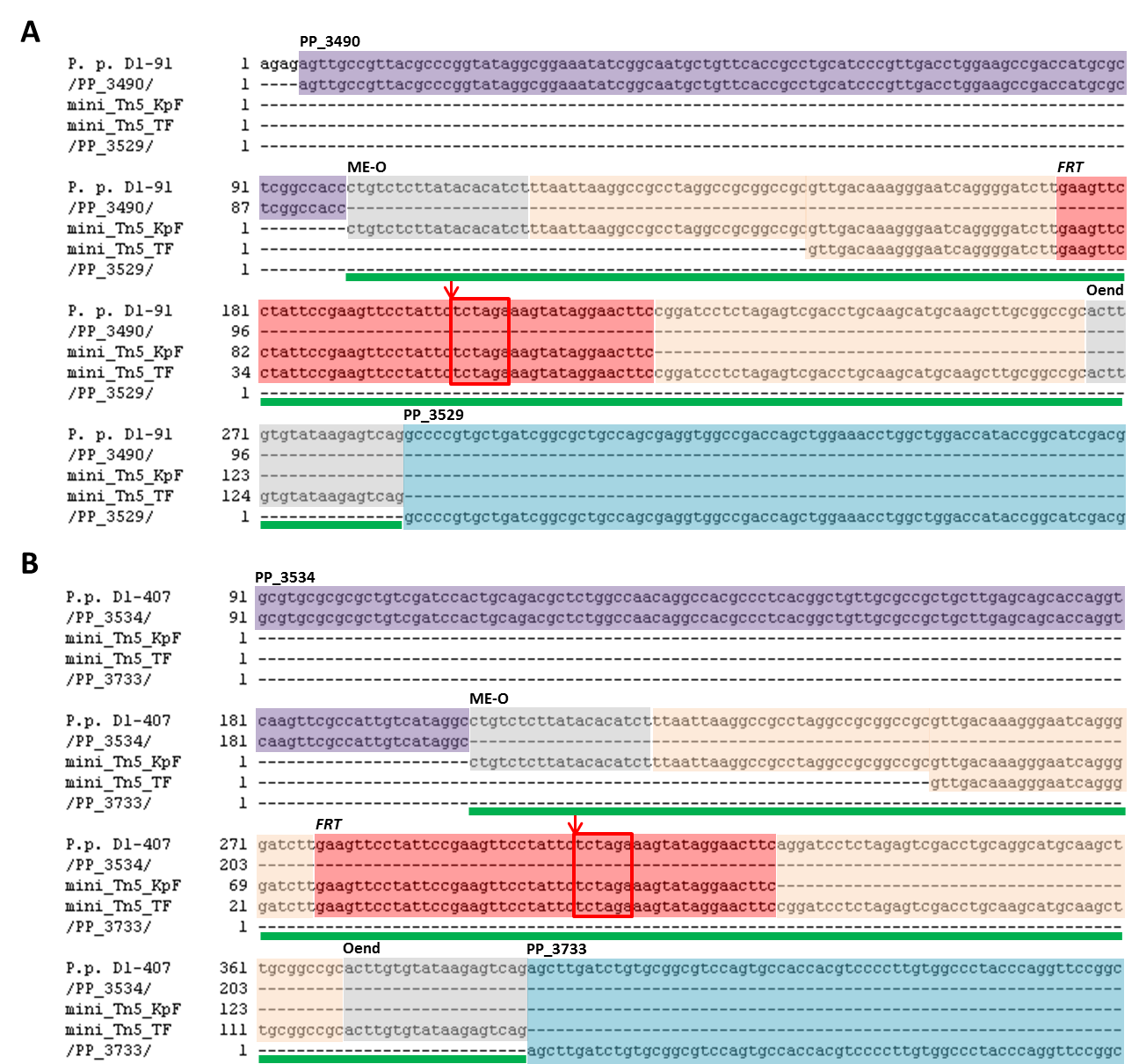


**Supplementary Figure S3:** **Verification of the deletion event**.

Amplification of the scar S1 (underlined in green) left after the single deletions and sequencing of the PCR products are presented here. (**A**) The scar, composed of the end sites (gray) and internal fragments (orange) of the mini-Tn*5* transposons and one *FRT* sequence (red), was formed in 91-∆_1_ mutant between the disrupted PP_3490 and PP_3529 genes. (**B**) the same scar was formed in 407-∆_1_ mutant between the disrupted PP_3534 and PP_3733 genes. The sequencing confirmed the absence of mutation in the *FRT* fragment, after alignment with the two mini-Tn*5* transposons in **A** and **B**. The red rectangle represents the recombination point between both *FRT* sites present in the genome of TMT mutant before deletion.

**Supplementary Table S1: Deleted genes in 91-∆_1_** mutant

| **Synonym** | ***Gene*** | **COG** | **Product** |
| --- | --- | --- | --- |
| **PP_3490** | *-* | - | hypothetical protein |
| **PP_3491** | *-* | COG1024I | enoly-coenzyme A hydratase/isomerase family protein |
| **PP_3492** | *acdA* | COG1960I | acyl-CoA dehydrogenase domain-containing protein |
| **PP_3493** | *-* | COG0583K | LysR family transcriptional regulator |
| **PP_3494** | *-* | - | hypothetical protein |
| **PP_3495** | *-* | COG2384R | hypothetical protein |
| **PP_3496** | *-* | COG0251J | endoribonuclease L-PSP |
| **PP_3497** | *-* | COG0826O | U32 family peptidase |
| **PP_3498** | *-* | COG3547L | ISPpu11, transposase |
| **PP_3499** | *-* | COG2963L | ISPpu14, transposase Orf1 |
| **PP_3500** | *-* | COG3436L | ISPpu14, transposase Orf2 |
| **PP_3501** | *-* | COG3436L | ISPpu14, transposase Orf3 |
| **PP_3502** | *-* | COG3547L | ISPpu10, transposase |
| **PP_3503** | *-* | COG2204T | Fis family sigma-54 specific transcriptional regulator |
| **PP_3504** | *-* | - | hypothetical protein |
| **PP_3505** | *-* | COG1240H | hypothetical protein |
| **PP_3506** | *-* | COG1239H | magnesium chelatase |
| **PP_3507** | *cobN* | COG1429H | cobaltochelatase subunit CobN |
| **PP_3508** | *cobW* | COG0523R | cobalamin biosynthesis protein CobW |
| **PP_3509** | *-* | - | glyoxalase family protein |
| **PP_3510** | *-* | - | hypothetical protein |
| **PP_3511** | *ilvE* | COG0115EH | branched-chain amino acid aminotransferase |
| **PP_3512** | *-* | COG4125S | transmembrane pair domain-containing protein |
| **PP_3513** | *-* | COG0583K | LysR family transcriptional regulator |
| **PP_3514** | *hyuB* | COG0146EQ | hydantoinase B/oxoprolinase |
| **PP_3515** | *hyuA* | COG0145EQ | 5-oxoprolinase |
| **PP_3516** | *-* | COG2207K | AraC family transcriptional regulator |
| **PP_3517** | *-* | - | hypothetical protein |
| **PP_3518** | *-* | - | hypothetical protein |
| **PP_3519** | *-* | - | lipoprotein |
| **PP_3520** | *-* | - | hypothetical protein |
| **PP_3521** | *-* | - | hypothetical protein |
| **PP_3522** | *-* | COG0251J | endoribonuclease L-PSP |
| **PP_3523** | *-* | - | hypothetical protein |
| **PP_3524** | *-* | - | hypothetical protein |
| **PP_3525** | *-* | - | hypothetical protein |
| **PP_3526** | *-* | COG4977K | AraC family transcriptional regulator |
| **PP_3527** | *-* | COG1309K | TetR family transcriptional regulator |
| **PP_3528** | *-* | COG0715P | aliphatic sulfonate ABC transporter periplasmic ligand-binding protein |
| **PP_3529** | *-* | COG2141C | monooxygenase |

**Supplementary Table S2: Deleted genes in 407-∆_1_ mutant**

| **Synonym** | ***Gene*** | **COG** | **Product** |
| --- | --- | --- | --- |
| **PP_3534** | *-* | COG0583K | LysR family transcriptional regulator |
| **PP_3535** | *ggt-1* | COG0405E | gamma-glutamyltransferase |
| **PP_3536** | *-* | - | hypothetical protein |
| **PP_3537** | *pobA* | COG0654HC | 4-hydroxybenzoate 3-monooxygenase |
| **PP_3538** | *pobR* | COG2207K | AraC family transcriptional regulator |
| **PP_3539** | *-* | COG0789K | transcriptional regulator |
| **PP_3540** | *mvaB* | COG0119E | hydroxymethylglutaryl-CoA lyase |
| **PP_3541** | *-* | COG1285S | Mg2+ transporter |
| **PP_3542** | *-* | - | hypothetical protein |
| **PP_3543** | *-* | COG0348C | iron-sulfur cluster-binding protein |
| **PP_3544** | *-* | COG1167KE | GntR family transcriptional regulator |
| **PP_3545** | *-* | COG0642T | PAS/PAC sensor hybrid histidine kinase |
| **PP_3546** | *-* | COG4191T | PAS/PAC sensor hybrid histidine kinase |
| **PP_3547** | *-* | COG1028IQR | short chain dehydrogenase/reductase family oxidoreductase |
| **PP_3548** | *-* | COG2814G | EmrB/QacA family drug resistance transporter |
| **PP_3549** | *emrA* | COG1566V | secretion protein HlyD family protein |
| **PP_3550** | *-* | COG1846K | MarR family transcriptional regulator |
| **PP_3551** | *-* | COG4566T | LuxR family DNA-binding response regulator |
| **PP_3552** | *-* | COG4191T | PAS/PAC sensor signal transduction histidine kinase |
| **PP_3553** | *-* | COG0318IQ | acyl-CoA synthetase |
| **PP_3554** | *-* | COG1960I | acyl-CoA dehydrogenase domain-containing protein |
| **PP_3555** | *-* | COG3712PT | anti-FecI sigma factor, FecR |
| **PP_3556** | *-* | COG2056R | Na+/H+ antiporter NhaC |
| **PP_3557** | *-* | COG0840NT | methyl-accepting chemotaxis transducer |
| **PP_3558** | *-* | COG2113E | substrate-binding region of ABC-type glycine betaine transport system |
| **PP_3559** | *-* | COG4176E | binding-protein-dependent transport systems inner membrane component |
| **PP_3560** | *-* | COG0583K | LysR family transcriptional regulator |
| **PP_3561** | *-* | COG0679R | auxin efflux carrier |
| **PP_3562** | *-* | COG3773M | hypothetical protein |
| **PP_3563** | *-* | COG2340S | hypothetical protein |
| **PP_3564** | *-* | COG4977K | AraC family transcriptional regulator |
| **PP_3565** | *-* | COG1280E | amino acid transporter LysE |
| **PP_3566** | *-* | COG2814G | major facilitator family transporter |
| **PP_3567** | *-* | COG0583K | LysR family transcriptional regulator |
| **PP_3568** | *-* | - | hypothetical protein |
| **PP_3569** | *-* | COG4993G | quinate dehydrogenase (pyrroloquinoline-quinone) |
| **PP_3570** | *-* | COG3659M | carbohydrate-selective porin OprB |
| **PP_3571** | *-* | COG0624E | acetylornithine deacetylase |
| **PP_3572** | *-* | COG3342S | hypothetical protein |
| **PP_3573** | *-* | COG2072P | monooxygenase |
| **PP_3574** | *-* | COG0251J | endoribonuclease L-PSP |
| **PP_3575** | *-* | COG1629P | TonB-dependent siderophore receptor |
| **PP_3576** | *-* | COG3712PT | anti-FecI sigma factor, FecR |
| **PP_3577** | *-* | COG1595K | ECF subfamily RNA polymerase sigma factor |
| **PP_3578** | *-* | COG0033G | phosphoglucomutase |
| **PP_3579** | *-* | COG4291S | hypothetical protein |
| **PP_3580** | *-* | - | hypothetical protein |
| **PP_3581** | *-* | COG5001T | GGDEF domain-containing protein |
| **PP_3582** | *-* | COG1538MU | RND efflux transporter |
| **PP_3583** | *-* | COG0841V | acriflavin resistance protein |
| **PP_3585** | *-* | COG0845M | efflux transporter RND family, MFP subunit |
| **PP_3586** | *-* | - | ISPpu9, transposase |
| **PP_3587** | *tpx* | COG2077O | redoxin domain protein |
| **PP_3588** | *-* | COG2814G | Bcr/CflA family multidrug resistance transporter |
| **PP_3589** | *sdaC* | COG0814E | aromatic amino acid ABC transporter permease |
| **PP_3590** | *tyrB-2* | COG1448E | aromatic amino acid aminotransferase |
| **PP_3591** | *-* | COG2055C | malate/L-lactate dehydrogenase |
| **PP_3592** | *-* | COG1737K | RpiR family transcriptional regulator |
| **PP_3593** | *-* | COG0834ET | amino acid ABC transporter periplasmic amino acid-binding protein |
| **PP_3594** | *-* | COG4215E | polar amino acid ABC transporter inner membrane subunit |
| **PP_3595** | *-* | COG4160E | polar amino acid ABC transporter inner membrane subunit |
| **PP_3596** | *-* | COG0665E | FAD dependent oxidoreductase |
| **PP_3597** | *-* | COG1126E | amino acid ABC transporter ATP-binding protein |
| **PP_3598** | *-* | COG2071R | peptidase C26 |
| **PP_3599** | *-* | COG0329EM | 5-dehydro-4-deoxyglucarate dehydratase |
| **PP_3600** | *-* | COG2271G | d-galactonate transporter |
| **PP_3601** | *-* | COG2721G | D-galactarate dehydratase |
| **PP_3602** | *-* | COG1012C | ketoglutarate semialdehyde dehydrogenase |
| **PP_3603** | *-* | COG2186K | GntR family transcriptional regulator |
| **PP_3604** | *-* | - | hypothetical protein |
| **PP_3605** | *-* | COG0583K | LysR family transcriptional regulator |
| **PP_3606** | *qor-2* | COG0604CR | quinone oxidoreductase |
| **PP_3607** | *-* | COG2391R | hypothetical protein |
| **PP_3608** | *-* | COG0583K | LysR family transcriptional regulator |
| **PP_3609** | *-* | COG3238S | hypothetical protein |
| **PP_3610** | *-* | COG4335L | hypothetical protein |
| **PP_3611** | *-* | - | hypothetical protein |
| **PP_3612** | *-* | COG4774P | TonB-dependent siderophore receptor |
| **PP_3613** | *-* | COG2133G | L-sorbosone dehydrogenase |
| **PP_3614** | *-* | COG4244S | hypothetical protein |
| **PP_3615** | *-* | - | hypothetical protein |
| **PP_3616** | *-* | - | hypothetical protein |
| **PP_3617** | *-* | - | hypothetical protein |
| **PP_3618** | *-* | - | hypothetical protein |
| **PP_3619** | *-* | - | hypothetical protein |
| **PP_3620** | *-* | COG1944S | hypothetical protein |
| **PP_3621** | *-* | COG2080C | (2Fe-2S)-binding domain protein |
| **PP_3622** | *-* | COG1529C | isoquinoline 1-oxidoreductase, beta subunit |
| **PP_3623** | *-* | COG2010C | gluconate 2-dehydrogenase acceptor subunit |
| **PP_3624** | *-* | COG0834ET | hypothetical protein |
| **PP_3625** | *-* | COG1280E | amino acid transporter LysE |
| **PP_3626** | *-* | COG3313R | hypothetical protein |
| **PP_3627** | *-* | - | hypothetical protein |
| **PP_3628** | *-* | COG1292M | choline/carnitine/betaine transporter |
| **PP_3629** | *-* | COG3865S | 3-demethylubiquinone-9 3-methyltransferase |
| **PP_3630** | *-* | - | porin |
| **PP_3631** | *-* | COG3103T | hypothetical protein |
| **PP_3632** | *-* | COG0583K | LysR family transcriptional regulator |
| **PP_3633** | *argC* | COG0002E | N-acetyl-gamma-glutamyl-phosphate reductase |
| **PP_3634** | *-* | - | acetyltransferase |
| **PP_3635** | *-* | COG0600P | binding-protein-dependent transport systems inner membrane component |
| **PP_3636** | *-* | COG0715P | sulfonate ABC transporter periplasmic sulfonate-binding protein |
| **PP_3637** | *-* | COG1116P | sulfonate ABC transporter ATP-binding protein |
| **PP_3638** | *-* | COG1960I | acyl-CoA dehydrogenase |
| **PP_3639** | *-* | COG2128S | alkylhydroperoxidase |
| **PP_3640** | *-* | COG2207K | AraC family transcriptional regulator |
| **PP_3641** | *-* | COG1457F | cytosine/purine/uracil/thiamine/allantoin permease family protein |
| **PP_3642** | *-* | - | hypothetical protein |
| **PP_3643** | *-* | COG1853R | oxidoreductase |
| **PP_3644** | *-* | COG2141C | luciferase family protein |
| **PP_3645** | *-* | COG0596R | alpha/beta fold family hydrolase |
| **PP_3646** | *-* | COG1012C | aldehyde dehydrogenase family protein |
| **PP_3647** | *-* | COG1853R | oxidoreductase |
| **PP_3648** | *-* | COG0599S | carboxymuconolactone decarboxylase |
| **PP_3649** | *-* | COG1802K | GntR family transcriptional regulator |
| **PP_3650** | *-* | COG1051F | NUDIX hydrolase |
| **PP_3651** | *-* | COG4191T | GAF sensor hybrid histidine kinase |
| **PP_3652** | *-* | COG1280E | amino acid transporter LysE |
| **PP_3653** | *-* | - | amino acid transporter LysE |
| **PP_3654** | *-* | COG1522K | AsnC family transcriptional regulator |
| **PP_3655** | *-* | COG1457F | cytosine/purine/uracil/thiamine/allantoin permease family protein |
| **PP_3656** | *-* | - | aromatic compound-specific porin |
| **PP_3657** | *-* | COG0778C | nitrobenzoate reductase |
| **PP_3658** | *-* | COG2271G | aromatic compound MFS transporter |
| **PP_3659** | *-* | COG2207K | AraC family transcriptional regulator |
| **PP_3660** | *-* | COG0583K | LysR family transcriptional regulator |
| **PP_3661** | *-* | COG2855S | hypothetical protein |
| **PP_3662** | *-* | COG1611R | decarboxylase family protein |
| **PP_3663** | *-* | COG2199T | GGDEF domain-containing protein |
| **PP_3664** | *pssA* | COG1502I | phosphatidylserine synthase |
| **PP_3665** | *-* | COG4977K | AraC family transcriptional regulator |
| **PP_3666** | *-* | COG2211G | major facilitator transporter |
| **PP_3667** | *-* | COG0006E | creatinase |
| **PP_3668** | *-* | COG0376P | catalase/peroxidase HPI |
| **PP_3669** | *-* | COG0583K | LysR family transcriptional regulator |
| **PP_3670** | *-* | - | hypothetical protein |
| **PP_3671** | *-* | COG0656R | aldo/keto reductase family oxidoreductase |
| **PP_3672** | *-* | COG2200T | hypothetical protein |
| **PP_3675** | *-* | - | hypothetical protein |
| **PP_3676** | *-* | - | hypothetical protein |
| **PP_3677** | *-* | - | hypothetical protein |
| **PP_3678** | *-* | - | hypothetical protein |
| **PP_3679** | *-* | COG0847L | hypothetical protein |
| **PP_3680** | *-* | COG3593L | hypothetical protein |
| **PP_3681** | *-* | COG0210L | helicase |
| **PP_3682** | *-* | - | hypothetical protein |
| **PP_3683** | *-* | - | hypothetical protein |
| **PP_3684** | *-* | - | hypothetical protein |
| **PP_3685** | *-* | - | hypothetical protein |
| **PP_3686** | *-* | - | hypothetical protein |
| **PP_3688** | *-* | - | hypothetical protein |
| **PP_3689** | *-* | - | serine/threonine protein phosphatase |
| **PP_3690** | *-* | - | hypothetical protein |
| **PP_3691** | *-* | COG1112L | DNA helicase-related protein |
| **PP_3692** | *-* | COG4988CO | hypothetical protein |
| **PP_3693** | *-* | - | transcriptional regulator MvaT, P16 subunit |
| **PP_3694** | *-* | - | hypothetical protein |
| **PP_3695** | *-* | COG4924S | hypothetical protein |
| **PP_3696** | *-* | COG4913S | hypothetical protein |
| **PP_3697** | *-* | COG4913S | hypothetical protein |
| **PP_3698** | *-* | - | hypothetical protein |
| **PP_3699** | *-* | COG2849S | hypothetical protein |
| **PP_3700** | *-* | COG1192D | hypothetical protein |
| **PP_3701** | *-* | - | hypothetical protein |
| **PP_3702** | *-* | - | hypothetical protein |
| **PP_3703** | *-* | - | hypothetical protein |
| **PP_3704** | *-* | - | hypothetical protein |
| **PP_3705** | *-* | - | hypothetical protein |
| **PP_3706** | *-* | COG0189HJ | hypothetical protein |
| **PP_3707** | *-* | - | hypothetical protein |
| **PP_3708** | *-* | - | hypothetical protein |
| **PP_3709** | *-* | - | hypothetical protein |
| **PP_3710** | *-* | - | hypothetical protein |
| **PP_3711** | *-* | COG5001T | sensory box protein |
| **PP_3712** | *-* | COG5628R | hypothetical protein |
| **PP_3713** | *catA* | COG3485Q | catechol 1,2-dioxygenase |
| **PP_3714** | *catC* | COG4829Q | muconolactone isomerase |
| **PP_3715** | *catB* | COG4948MR | muconate and chloromuconate cycloisomerase |
| **PP_3716** | *catR* | COG0583K | LysR family transcriptional regulator |
| **PP_3717** | *-* | COG2771K | LuxR family transcriptional regulator |
| **PP_3718** | *-* | COG0161H | hypothetical protein |
| **PP_3719** | *-* | COG0687E | periplasmic polyamine-binding protein |
| **PP_3720** | *-* | COG2249R | ribosyldihydronicotinamide dehydrogenase (quinone) |
| **PP_3721** | *aspC* | COG0436E | aspartate aminotransferase |
| **PP_3722** | *alr* | COG0787M | alanine racemase |
| **PP_3724** | *-* | COG1042C | acyl-CoA synthetase |
| **PP_3725** | *-* | COG1960I | acyl-CoA dehydrogenase domain-containing protein |
| **PP_3726** | *-* | COG1024I | enoyl-CoA hydratase/isomerase |
| **PP_3727** | *-* | COG0833E | amino acid transporter |
| **PP_3728** | *-* | COG0642T | multi-sensor hybrid histidine kinase |
| **PP_3729** | *-* | COG0834ET | periplasmic amino acid-binding protein-related protein |
| **PP_3730** | *-* | COG0745TK | winged helix family two component transcriptional regulator |
| **PP_3731** | *-* | COG1309K | TetR family transcriptional regulator |
| **PP_3732** | *-* | COG1024I | enoyl-CoA hydratase/isomerase |
| **PP_3733** | *-* | COG5621R | ABC transporter |

**Supplementary Table S3: Second deletion in 407.1-∆_2_** mutant

| **Synonym** | ***Gene*** | | | **COG** | **Product** |
| --- | --- | --- | --- | --- | --- |
| **PP_4290** | | *-* | COG2233F | | xanthine/uracil permease family protein |
| **PP_4291** | | *-* | COG3248M | | hypothetical protein |
| **PP_4292** | | *-* | - | | hypothetical protein |
| **PP_4293** | | *-* | COG3248M | | hypothetical protein |
| **PP_4294** | | *-* | COG2354S | | hypothetical protein |
| **PP_4295** | | *-* | COG1309K | | TetR family transcriptional regulator |
| **PP_4296** | | *-* | COG3193R | | hypothetical protein |
| **PP_4297** | | *gcl* | COG3960R | | glyoxylate carboligase |
| **PP_4298** | | *hyi* | COG3622G | | hydroxypyruvate isomerase |
| **PP_4299** | | *glxR* | COG2084I | | 2-hydroxy-3-oxopropionate reductase |
| **PP_4300** | | *-* | COG2379G | | hydroxypyruvate reductase |
| **PP_4301** | | *pykF* | COG0469G | | pyruvate kinase |
| **PP_4302** | | *-* | COG4413E | | Urea transporter |
| **PP_4303** | | *-* | - | | hypothetical protein |
| **PP_4304** | | *-* | COG1226P | | cation transporter voltage-gated ion channel cation transporter |
| **PP_4305** | | *-* | COG1613P | | sulfate ABC transporter periplasmic sulfate-binding protein |
| **PP_4306** | | *-* | COG1092R | | hypothetical protein |
| **PP_4307** | | *-* | COG2962R | | hypothetical protein |
| **PP_4308** | | *-* | COG1522K | | AsnC family transcriptional regulator |

**Supplementary Table S4: Second deletion in 407.3-∆_2_ mutant**

| **Synonym** | ***Gene*** | | | **COG** | **Product** |
| --- | --- | --- | --- | --- | --- |
| **PP_3360** | | *-* | COG0668M | | hypothetical protein |
| **PP_3361** | | *-* | COG0160E | | hypothetical protein |
| **PP_3362** | | *-* | COG1522K | | AsnC family transcriptional regulator |
| **PP_3363** | | *-* | COG0125F | | hypothetical protein |
| **PP_3364** | | *-* | COG4251T | | response regulator |
| **PP_3365** | | *-* | COG0028EH | | acetolactate synthase |
| **PP_3366** | | *-* | COG0583K | | LysR family transcriptional regulator |
| **PP_3367** | | *-* | COG2945R | | hypothetical protein |
| **PP_3368** | | *-* | COG2814G | | major facilitator family transporter |
| **PP_3369** | | *-* | COG0583K | | LysR family transcriptional regulator |
| **PP_3370** | | *-* | COG0667C | | aldo/keto reductase family oxidoreductase |
| **PP_3371** | | *-* | COG0642T | | integral membrane sensor signal transduction histidine kinase |
| **PP_3372** | | *cpxR* | COG0745TK | | winged helix family two component transcriptional regulator |
| **PP_3373** | | *-* | COG4775M | | surface antigen family protein |
| **PP_3374** | | *-* | - | | hypothetical protein |
| **PP_3375** | | *endA-2* | COG2356L | | deoxyribonuclease I |
| **PP_3376** | | *kguD* | COG1052CHR | | 2-ketogluconate 6-phosphate reductase |
| **PP_3377** | | *-* | COG2271G | | major facilitator transporter |
| **PP_3378** | | *kguK* | COG0524G | | PfkB domain protein |
| **PP_3379** | | *-* | - | | xylose isomerase |
| **PP_3380** | | *ptxS* | COG1609K | | PtxS family transcriptional regulator |
| **PP_3381** | | *-* | - | | ISPpu9, transposase |
| **PP_3382** | | *-* | COG2010C | | gluconate 2-dehydrogenase acceptor subunit |
| **PP_3383** | | *-* | COG2303E | | gluconate 2-dehydrogenase acceptor subunit |
| **PP_3384** | | *-* | - | | gluconate 2-dehydrogenase acceptor subunit |
| **PP_3385** | | *-* | COG3455S | | hypothetical protein |
| **PP_3386** | | *vgrG-2* | COG3501S | | vgrG protein |
| **PP_3387** | | *-* | - | | hypothetical protein |
| **PP_3388** | | *-* | - | | hypothetical protein |
| **PP_3389** | | *-* | - | | hypothetical protein |
| **PP_3390** | | *-* | - | | porin |
| **PP_3391** | | *-* | COG2271G | | tartrate MFS transporter |
| **PP_3392** | | *-* | COG0663R | | anhydrase family 3 protein |
| **PP_3393** | | *-* | COG1804C | | CAIB/BAIF family protein |
| **PP_3394** | | *-* | COG0119E | | 3-hydroxy-3-methylglutaryl-CoA lyase |
| **PP_3395** | | *-* | COG0583K | | LysR family transcriptional regulator |
| **PP_3396** | | *-* | - | | hypothetical protein |
| **PP_3397** | | *-* | - | | hypothetical protein |
| **PP_3398** | | *-* | - | | curlin associated repeat protein |
| **PP_3400** | | *alkB* | COG3145L | | 2OG-Fe(II) oxygenase |
| **PP_3401** | | *-* | - | | hypothetical protein |
| **PP_3402** | | *-* | - | | hypothetical protein |
| **PP_3403** | | *-* | COG1741R | | Pirin domain protein |
| **PP_3404** | | *-* | COG0596R | | alpha/beta hydrolase fold |
| **PP_3405** | | *-* | COG1280E | | lysine exporter protein LysE/YggA |
| **PP_3406** | | *-* | COG1247M | | acetyltransferase |
| **PP_3407** | | *-* | - | | hypothetical protein |
| **PP_3409** | | *-* | COG2073H | | cobalamin biosynthesis protein cobE |
| **PP_3410** | | *cobM* | COG2875H | | precorrin-4 C11-methyltransferase |
| **PP_3411** | | *-* | COG3813S | | hypothetical protein |
| **PP_3412** | | *-* | COG2197TK | | LuxR family DNA-binding response regulator |
| **PP_3413** | | *-* | COG0642T | | Hpt sensor hybrid histidine kinase |
| **PP_3414** | | *-* | COG0840NT | | methyl-accepting chemotaxis transducer/sensory box protein |
| **PP_3415** | | *-* | COG1609K | | LacI family transcription regulator |
| **PP_3416** | | *gnuK* | COG3265G | | carbohydrate kinase, thermoresistant glucokinase family |
| **PP_3417** | | *gntP* | COG2610GE | | gluconate transporter |
| **PP_3418** | | *-* | - | | hypothetical protein |
| **PP_3419** | | *-* | COG2204T | | two component, sigma54 specific, transcriptional regulator, Fis family |
| **PP_3420** | | *-* | COG0642T | | sensor histidine kinase |
| **PP_3421** | | *-* | COG0642T | | sensor histidine kinase |
| **PP_3422** | | *ltg* | COG0741M | | lytic transglycosylase |
| **PP_3423** | | *xcpT-2* | COG2165NU | | general secretion pathway protein G |
| **PP_3424** | | *xcpS-2* | COG1459NU | | type II secretion system protein |
| **PP_3425** | | *mexE* | COG0845M | | efflux transporter RND family, MFP subunit |
| **PP_3426** | | *mexF* | COG0841V | | transporter hydrophobe/amphiphile efflux-1 (HAE1) family |
| **PP_3427** | | *oprN* | COG1538MU | | RND efflux system, outer membrane lipoprotein, NodT family |
| **PP_3428** | | *-* | COG3063NU | | hypothetical protein |
| **PP_3429** | | *-* | COG0642T | | histidine kinase |
| **PP_3430** | | *-* | COG0642T | | PAS/PAC sensor hybrid histidine kinase |
| **PP_3431** | | *-* | COG0693R | | ThiJ/PfpI domain protein |
| **PP_3432** | | *-* | - | | hypothetical protein |
| **PP_3433** | | *hpd* | COG3185ER | | 4-hydroxyphenylpyruvate dioxygenase |
| **PP_3434** | | *-* | - | | hypothetical protein |
| **PP_3435** | | *-* | COG2200T | | diguanylate phosphodiesterase |
| **PP_3436** | | *rarD-2* | COG2962R | | RarD protein, DMT superfamily transporter |
| **PP_3437** | | *-* | COG0861P | | CBS domain-containing protein |
| **PP_3438** | | *-* | COG1280E | | lysine exporter protein LysE/YggA |
| **PP_3439** | | *-* | COG2207K | | AraC family transcriptional regulator |
| **PP_3440** | | *-* | - | | hypothetical protein |
| **PP_3441** | | *-* | - | | hypothetical protein |
| **PP_3442** | | *-* | - | | hypothetical protein |
| **PP_3443** | | *-* | COG1012C | | glyceraldehyde-3-phosphate dehydrogenase |
| **PP_3444** | | *-* | COG2764S | | glyoxalase/bleomycin resistance protein/dioxygenase |
| **PP_3445** | | *-* | - | | hypothetical protein |
| **PP_3446** | | *ilvA-1* | COG1171E | | threonine dehydratase |
| **PP_3447** | | *-* | COG4125S | | transmembrane pair domain protein |
| **PP_3448** | | *nfrB* | COG1215M | | bacteriophage N4 adsorption protein B |
| **PP_3449** | | *-* | - | | hypothetical protein |
| **PP_3450** | | *-* | - | | hypothetical protein |
| **PP_3451** | | *-* | - | | hypothetical protein |
| **PP_3452** | | *-* | COG3706T | | GGDEF domain-containing protein |
| **PP_3453** | | *-* | COG0642T | | integral membrane sensor signal transduction histidine kinase |
| **PP_3454** | | *-* | COG0745TK | | winged helix family two component transcriptional regulator |
| **PP_3455** | | *-* | COG0845M | | efflux transporter RND family, MFP subunit |
| **PP_3456** | | *-* | COG0841V | | transporter hydrophobe/amphiphile efflux-1 (HAE1) family |
| **PP_3457** | | *mobA* | COG0746H | | molybdopterin-guanine dinucleotide biosynthesis protein MobA |
| **PP_3458** | | *-* | COG0318IQ | | acyl-CoA synthetase |
| **PP_3459** | | *-* | COG3391S | | hypothetical protein |
| **PP_3460** | | *-* | - | | hypothetical protein |
| **PP_3461** | | *-* | COG0641R | | radical SAM domain protein |
| **PP_3462** | | *-* | - | | hypothetical protein |
| **PP_3463** | | *-* | COG1012C | | aldehyde dehydrogenase |
| **PP_3464** | | *-* | - | | hypothetical protein |
| **PP_3465** | | *-* | - | | hypothetical protein |
| **PP_3466** | | *-* | COG1132V | | efflux ABC transporter ATP-binding protein |
| **PP_3467** | | *-* | COG3284QK | | GAF modulated sigma54 specific transcriptional regulator, Fis family |
| **PP_3468** | | *-* | - | | hypothetical protein |
| **PP_3469** | | *-* | - | | hypothetical protein |
| **PP_3471** | | *-* | - | | hypothetical protein |
| **PP_3472** | | *csgG* | COG1462M | | curli production assembly/transport component CsgG |
| **PP_3473** | | *-* | - | | curli fiber protein CsgF |
| **PP_3474** | | *-* | - | | curli assembly protein CsgE |
| **PP_3475** | | *-* | COG2165NU | | hypothetical protein |
| **PP_3476** | | *-* | COG2165NU | | type II secretion system protein G |
| **PP_3477** | | *-* | COG2165NU | | hypothetical protein |
| **PP_3478** | | *-* | COG4796U | | type II and III secretion system protein |
| **PP_3479** | | *-* | - | | hypothetical protein |
| **PP_3480** | | *-* | - | | hypothetical protein |
| **PP_3481** | | *-* | - | | hypothetical protein |
| **PP_3482** | | *-* | - | | hypothetical protein |
| **PP_3483** | | *-* | COG2804NU | | type II secretion system protein E |
| **PP_3484** | | *-* | COG2204T | | response regulator receiver protein |
| **PP_3485** | | *-* | - | | hypothetical protein |
| **PP_3486** | | *-* | COG0683E | | cytochrome c |
| **PP_3487** | | *-* | COG1999R | | electron transport protein SCO1/SenC |
| **PP_3488** | | *-* | COG1999R | | electron transport protein SCO1/SenC |
| **PP_3489** | | *-* | - | | hypothetical protein |
| **PP_3490** | | *-* | - | | hypothetical protein |
| **PP_3491** | | *-* | COG1024I | | enoly-coenzyme A hydratase/isomerase family protein |
| **PP_3492** | | *acdA* | COG1960I | | acyl-CoA dehydrogenase domain-containing protein |
| **PP_3493** | | *-* | COG0583K | | LysR family transcriptional regulator |
| **PP_3494** | | *-* | - | | hypothetical protein |
| **PP_3495** | | *-* | COG2384R | | hypothetical protein |
| **PP_3496** | | *-* | COG0251J | | endoribonuclease L-PSP |
| **PP_3497** | | *-* | COG0826O | | U32 family peptidase |
| **PP_3498** | | *-* | COG3547L | | ISPpu11, transposase |
| **PP_3499** | | *-* | COG2963L | | ISPpu14, transposase Orf1 |
| **PP_3500** | | *-* | COG3436L | | ISPpu14, transposase Orf2 |
| **PP_3501** | | *-* | COG3436L | | ISPpu14, transposase Orf3 |
| **PP_3502** | | *-* | COG3547L | | ISPpu10, transposase |
| **PP_3503** | | *-* | COG2204T | | sigma54 specific transcriptional regulator, Fis family |
| **PP_3504** | | *-* | - | | hypothetical protein |
| **PP_3505** | | *-* | COG1240H | | hypothetical protein |
| **PP_3506** | | *-* | COG1239H | | magnesium chelatase |
| **PP_3507** | | *cobN* | COG1429H | | cobaltochelatase subunit CobN |
| **PP_3508** | | *cobW* | COG0523R | | cobalamin biosynthesis protein CobW |
| **PP_3509** | | *-* | - | | glyoxalase family protein |
| **PP_3510** | | *-* | - | | hypothetical protein |
| **PP_3511** | | *ilvE* | COG0115EH | | branched-chain amino acid aminotransferase |
| **PP_3512** | | *-* | COG4125S | | transmembrane pair domain protein |
| **PP_3513** | | *-* | COG0583K | | LysR family transcriptional regulator |
| **PP_3514** | | *hyuB* | COG0146EQ | | hydantoinase B/oxoprolinase |
| **PP_3515** | | *hyuA* | COG0145EQ | | 5-oxoprolinase (ATP-hydrolyzing) |
| **PP_3516** | | *-* | COG2207K | | AraC family transcriptional regulator |
| **PP_3517** | | *-* | - | | hypothetical protein |
| **PP_3518** | | *-* | - | | hypothetical protein |
| **PP_3519** | | *-* | - | | lipoprotein |
| **PP_3520** | | *-* | - | | hypothetical protein |
| **PP_3521** | | *-* | - | | hypothetical protein |
| **PP_3522** | | *-* | COG0251J | | endoribonuclease L-PSP |
| **PP_3523** | | *-* | - | | hypothetical protein |
| **PP_3524** | | *-* | - | | hypothetical protein |
| **PP_3525** | | *-* | - | | hypothetical protein |
| **PP_3526** | | *-* | COG4977K | | AraC family transcriptional regulator |
| **PP_3527** | | *-* | COG1309K | | TetR family transcriptional regulator |
| **PP_3528** | | *-* | COG0715P | | aliphatic sulfonate ABC transporter periplasmic ligand-binding protein |
| **PP_3529** | | *-* | COG2141C | | monooxygenase |
| **PP_3530** | | *-* | COG3257R | | hypothetical protein |
| **PP_3531** | | - | - | | lipid-binding START domain protein |
| **PP_3532** | | - | COG1522K | | AsnC family transcriptional regulator |
| **PP_3533** | | - | COG2423E | | ornithine cyclodeaminase |

**Supplementary Table S**5: Primers for the customized mini-transposons

| **Name of primer** | **Target** | **Sequence (5’-3’)** |
| --- | --- | --- |
| *pyrF* 1F | *pyrF* | cgGGATCCGTCGGGCCGTGGGCAACG |
| *pyrF* 2R | *pyrF* | cccgAAGCTTTTACCCACGGATCTCCGC |
| Fp 1F | *FRT*::*pyrF* | ataagaatGCGGCCGCGTTGACAAAGGGAATCAGGG |
| Fp 2R | *FRT*::*pyrF* | ataagaatGCGGCCGCAAGCTTTTAC |
| F 10F | *FRT* | ATTCCGGGCATTGCTGTTGAC |
| F 11R | *FRT* | GGCCTGGCACCTATAATTGAACC |

**Supplementary Table S6: Primers for Southern blot experiments**

| **Name of primer** | **Target** | **Sequence (5’-3’)** |
| --- | --- | --- |
| Km-pBAM-F | *pyrF* | TATTCAGCGTGAAACGAGCTGTAG |
| Km-pBAM-R | *pyrF* | CGGATTATCAATGCCATATTTCTG |
| Tel-F | *FRT*::*pyrF* | GAAGCAGGCGAGAAACTGAC |
| Tel-R | *FRT*::*pyrF* | TTGATGAGCGTCGTCTGAAC |

**Supplementary Table S7: Primers for Arbitrary Primed-PCR experiments**

| **Name of primer** | **Target** | **Round** | **Sequence (5’-3’)** |
| --- | --- | --- | --- |
| P1 |  |  |  |
| APPCR-Km1 | mini-Tn*5 KpF* | 1^st^ | CTACAGCTCGTTTCACGCTGAATA |
| kilA-rev | mini-Tn*5 TF* | 1^st^ | ACGCTTTGTTCTTCCATTCG |
| P2 |  |  |  |
| APPCR-Km3 | mini-Tn*5 KpF* | 2^nd^ | CTTGTGCAATGTAACATCAGAG |
| F 10F | mini-Tn*5 TF* | 2^nd^ | attcCGGGCATTGCTGTTGAC |
| ARB1 |  | 1^st^ | GGCCACGCGTCGACTAGTACNNNNNNNNNNGATAT |
| ARB2 |  | 2^nd^ | GGCCACGCGTCGACTAGTAC |

1st: primer used in the first round of PCR

2nd: primer used in the second round of PCR
